# Pili and other surface proteins influence the structure and the nanomechanical properties of *Lactococcus lactis* biofilms

**DOI:** 10.1038/s41598-021-84030-1

**Published:** 2021-03-01

**Authors:** Ibrahima Drame, Christine Lafforgue, Cecile Formosa-Dague, Marie-Pierre Chapot-Chartier, Jean-Christophe Piard, Mickaël Castelain, Etienne Dague

**Affiliations:** 1 TBI, Université de Toulouse, INSA, INRAE, CNRS, Toulouse, France; 2 LAAS-CNRS, Université de Toulouse CNRS, Toulouse, France; 3 Université Paris-Saclay, INRAE, AgroParisTech, Micalis Institute, 78350 Jouy-en-Josas, France; 4 Fédération de Recherche Fermat, CNRS, Toulouse, France

**Keywords:** Nanoscale biophysics, Biofilms

## Abstract

Lactic acid bacteria, in particular *Lactococcus lactis*, are widely used in the food industry, for the control and/or the protection of the manufacturing processes of fermented food. While *L. lactis* has been reported to form compact and uniform biofilms it was recently shown that certain strains able to display pili at their surface form more complex biofilms exhibiting heterogeneous and aerial structures. As the impact of those biofilm structures on the biomechanical properties of the biofilms is poorly understood, these were investigated using AFM force spectroscopy and imaging. Three types of strains were used *i.e.,* a control strain devoid of pili and surface mucus-binding protein, a strain displaying pili but no mucus-binding proteins and a strain displaying both pili and a mucus-binding protein. To identify potential correlations between the nanomechanical measurements and the biofilm architecture, 24-h old biofilms were characterized by confocal laser scanning microscopy. Globally the strains devoid of pili displayed smoother and stiffer biofilms (Young Modulus of 4–100 kPa) than those of piliated strains (Young Modulus around 0.04–0.1 kPa). Additional display of a mucus-binding protein did not affect the biofilm stiffness but made the biofilm smoother and more compact. Finally, we demonstrated the role of pili in the biofilm cohesiveness by monitoring the homotypic adhesion of bacteria to the biofilm surface. These results will help to understand the role of pili and mucus-binding proteins withstanding external forces.

## Introduction

Bacterial biofilms are complex communities, growing on a surface and embedded in a self-produced extracellular polymeric matrix^1–4^. Extracellular polymeric substances (EPS) are composed of proteins, extracellular DNA, and mainly polysaccharides. They are considered as key components that determine the physicochemical and biological properties of biofilms and contribute to their mechanical stability^4,5^. Bacterial biofilms have been extensively studied since the 1970s, particularly biofilms of pathogenic bacteria. Indeed, they have been described to be responsible for different issues including persistent infections^6^, antibiotic resistance^7^ and quality/safety problems in food industry^8^.

On the other hand, beneficial biofilms formed by non-pathogenic bacteria have received increasing interest over the last 10 years. Bacteria such as the probiotic *Lactobacillus rhamnosus* GG^9,10^, the commensal bifidobacteria^11^ or the food lactic acid bacterium *Lactococcus lactis*^12–15^, have been increasingly studied for their implication in the safety of food fermented products and in their probiotic properties. Among these bacterial species, the archetypal lactic acid bacterium *L. lactis* has been studied for its capacity to adhere and to form biofilm on biotic and abiotic surfaces^13,16–18^. *L. lactis* is a Gram-positive bacterial species living in nutrient-rich ecological niches (plants, gut mucus and milk) and is the most widely used bacteria in dairy industry for cheese or lactic products manufacturing^19,20^. *L. lactis* can be used to form beneficial biofilms that have the potential to prevent and control food spoilage induced by pathogenic bacteria in food processing environments^8,21^. In addition, *L. lactis* has been proposed as a potent candidate in biotechnological applications as cell factories organism and as delivery vehicles of beneficial molecules (antigens and cytokines) in the development of live mucosal vaccines^22,23^.

To better exploit *L. lactis* biofilms, it is essential to improve the description and the understanding of their behaviors and the relation between their structuration (adhesion, cohesion, and organization) and their function. While the role of surface proteins and appendages such as pili in the mechanisms of single cell adhesion to a surface^24^ or of cell–cell interactions have been studied^25^, the global biomechanical properties of the entire biofilm are still largely ignored. In a previous study^25^ we have demonstrated and described, at the molecular scale, the role of pili in homotypic interactions between *L. lactis* cells. Indeed, applying the single cell force spectroscopy technic, we showed that cell–cell adhesion abilities were driven by the presence of pili on their surface. Both laboratory and environmental *L. lactis* showed a high adhesion force and work, in the case of piliated strains whereas strains devoid of pili showed weak interactions. The objective of the present work is to go further and establish the role and functions of pili in the structuration and organization of the biofilm. We aim at linking our previous findings (pili–pili molecular and homotypic interactions between cells) with their consequences at the biofilm level. Pili are crucial for bacterial cell adhesion to a solid surface that is the first step of biofilm formation, but pili are likely to play a role also in the nanomechanical properties of the formed biofilms. Thus, the understanding of the complex microstructuration of biofilms is essential if we are to control them^26^. Such a role of pili has been widely studied in the biofilms formed by pathogenic bacteria. For example, *Pseudomonas aeruginosa*, a model organism used to study pathogenic biofilms is able to form biofilms with spatial structures^27–29^ because it displays type IV pili at its surface^30^. Type IV pili have been shown, by confocal laser scanning microscopy (CLSM), to play a role in cell aggregation, micro-colony formation and biofilm differentiation^30^. They are also involved in the formation of heterogeneous biofilms showing mushroom-shaped multicellular structures^28,31^. In another study, Yi *and al*.^32^ investigated the role of pili in the formation of biofilm by the human pathogen *Neisseria meningitidis*. This study showed that pili and EPS of *N. meningitidis* are involved both in the attachment of cells to a suitable surface and in the biofilm architecture^32^. Finally, in Gram-positive pathogens such as *Enterococcus faecalis*^33^ or *Streptococcus pyogenes*^34^, the importance of pili in biofilm development was highlighted in studies where the authors demonstrated that pili-devoid strains were defective in binding to epithelial cells and presented prominent defects in biofilm formation. More recently, pili have been investigated in non-pathogenic Gram-positive bacteria, including lactic acid bacteria such as *Lactobacillus rhamnosus* GG^35^ and *L. lactis*^14,36^. Pili of *L. rhamnosus* GG cells allow to establish both long and short distance contacts with host tissues^37^ thus allowing to strengthen adhesion and to withstand shear stresses in natural environments.

Despite the high potential of *L. lactis* to form biofilms on abiotic and biotic surfaces, the nanomechanical properties of these biofilms and the influence of pili on their structure and porosity properties are still poorly understood. A study by Oxaran and co-workers^14^ reported that the model laboratory strain *L. lactis* IL1403 usually forms compact and uniform biofilms. However, when isogenic *L. lactis* IL1403 cells displayed pili, the formed biofilms were highly reticulated and appeared heterogeneous, rough and aerial^14^. The authors also demonstrated that pili have a key role in auto-aggregation phenotypes and in the formation of thicker biofilms than those observed with pilus-deficient wild-type strains (85 µm versus 45 µm). While biofilms formed by *L. lactis* strain IL1403 and its isogenic derivatives have been studied, no studies have yet been carried out on the biofilms formed by the environmental isolate *L. lactis* TIL448 that is natively able to display pili. In this strain, the genes required for pilus biogenesis are harbored in a plasmid and the backbone pilin (YhgE2) exhibits 28% of amino acid sequence similarity with YhgE of *L. lactis* IL1403^38^. The pili were shown to be involved in adhesion of TIL448 strain to biotic surfaces including Caco-2 intestinal epithelial cells^38^ and mucins^38,39^. Another difference between *L. lactis* IL1403 and TIL448 is the ability of the latter to harbor a plasmid-borne gene for a mucin-binding protein (Mub) involved in adhesion to mucins^39^.

The presence of pili at the cell surface is therefore involved in the biofilm structure and most probably plays a role in the architecture and the mechanical properties of biofilms. The organization of biofilms and their surface morphology have often been investigated by confocal laser scanning microscopy (CLSM) or transmission electron microscopy (TEM), and more recently atomic force microscopy (AFM)^40^ which is powerful to get information at the molecular level. To investigate the mechanical properties of biofilms, specific nanoindentation measurements^41^ as well as phonatory rheometry^42^ can be used to characterize the bulk properties. Such techniques are however limited because of their inability to measure the properties of intact, hydrated biofilms on native surfaces. In recent years, AFM techniques^43,44^ offered the possibility to characterize the topology and roughness of a biofilm surface^45^ and to access its mechanical properties^46^, in an aqueous environment. AFM also becomes the most common and efficient technique to understand the force involved in cell adhesion and biofilm cohesiveness^47–49^.

In a previous work^25^, we have investigated the role of pili in the interactions between bacterial cells and demonstrated that piliated cells were more likely to interact with each other compared to non-piliated cells. In the present work, our aim is to investigate (i) the role of pili in the structure and the organization of *L. lactis* biofilm and (ii) the relation between its nanomechanical properties and its architecture. To reach this goal, CLSM was used to analyze the biofilm structure combined with AFM to image at high resolution the biofilm surface topology and to measure its nanomechanical properties. The interactions between cells immobilized on an AFM colloidal probe and 24-h old biofilms were also measured to demonstrate the role of pili in cells adhesion to more or less cohesive biofilms. Studying the role of pili in *L. lactis* biofilms should allow to better understand their adhesion and persistence in environments such as the human gut or the dairy industry in which this bacteria occurs and may control the development of pathogenic or spoilage species.

## Materials and methods

### *L. lactis* strains and their growth conditions

Bacterial strains derived from *L. lactis subsp. lactis* used in this study are described in Table 1. Strains were grown in M17 broth (Oxoid) medium containing 0.5% (w/v) of D-glucose (M17 Glc). When required, erythromycin (Ery) and/or tetracycline (Tet) were added to the medium to a final concentration of 5 µg/mL. The cultures were incubated overnight at 30 °C under static conditions as described elsewhere^25^.

**Table 1:** * L. lactis* strains used in this study.

Strains	Genotype or phenotype	Source/reference
VE17061	Pil^−^, control (pili minus)	^14^
VE17176	Pil^++^, over expression of both the *pil* operon and the *srtA* gene	^14^
TIL448	Pil^+^/Mub^+^, vegetal isolate from peas	^38^
TIL1230	Pil^−^/Mub^−^, TIL448 derivative obtained by plasmid curing	^38^

### Biofilm culture

First, 3 mL of phosphate buffered saline (PBS, 1X) solution containing 4 mg/mL of dopamine hydrochloride (99%, Sigma) were introduced in Culture-treated Petri dish (TPP, ø × h = 40 × 11 mm) and incubated for 1 h under sterile conditions. After removing the dopamine hydrochloride solution from the Petri dish, a droplet of 50 µL of overnight culture was deposited in the Petri dish containing 3 mL of culture medium. After 24-h of growth at 30 °C under static conditions and formation of the biofilm, the old medium was removed and replaced by fresh PBS to conduct AFM experiments.

### Confocal laser scanning microscopy (CLSM)

To analyze the biofilm structure and thickness, 24-h biofilm-embedded cells in PBS were stained with Syto-9 dye from the *BacLight* bacterial viability kit (Invitrogen, Cergy Pontoise, France) and observed with Confocal Leica DMR TCS SP2 (Leica microsystems, Wetzlar, Germany, with magnification of 10×) fitted with water immersion dipping lenses. The excitation wavelength at 488 nm (blue laser) generated green fluorescence and all light rays emitted above 500 nm were collected. Biofilm structure was analyzed by taking a series of horizontal sections (stacks) to evaluate its thickness. All images were processed using Leica Confocal Software Lite.

### AFM imaging and force measurements

To analyze the surface topography of biofilms from each *L. lactis* strain, AFM images of an area of 50 µm × 50 µm (JPK Instruments, Bruker, USA) were recorded in contact mode in PBS at room temperature. MLCT cantilevers (Bruker, USA, nominal spring constant of ~ 0.01 N/m, as determined using the thermal noise method^50^) were used with a scanning rate of 1 Hz and a resolution of 128 × 128 pixels. The images acquired were analyzed using the Data Processing software from JPK Instruments (Bruker, USA).

Mechanical properties of the different *L. lactis* biofilms were measured in force spectroscopy experiments by recording a grid map of 16-by-16 force curves on an area of 50 µm × 50 µm in PBS. For that, AFM colloidal probes were used and prepared using the protocol described in^51^. Briefly, colloidal probes were obtained by attaching a single silica microsphere (5 μm diameter, Bangs Laboratories) with a thin layer of UV-curable glue (NOA 63, Norland Edmund Optics) on triangular tipless cantilevers (NP-O10, Bruker, USA) and using a Nanowizard III AFM (Bruker USA). The nominal spring constant of the colloidal probe cantilever was 0.04–0.08 N/m as determined using the thermal noise method^50^. The maximal applied force was 0.5 nN for each condition, the constant approach/retract speed of 12 to 30 µm/s and the z-length of the piezo was adjusted up to 15 µm. Force curves obtained were then converted into indentation curves and fitted to the Hertz model to obtain Young’s modulus values. The Hertz model for spherical indentation follows the equation *F*_*cant*_ = *4/3 *(E*R^1*/*2^*δ*^3/2^*)*, where F is the nanoindention force applied, E* is the reduced Young’s modulus, *δ* is the deformation of the sample in contact, and *R* is the spherical colloidal probe radius, taken as 2.5 µm.

To measure the adhesion forces between single cells and biofilms, AFM colloidal probes were first immersed for 1 h in 50 µL of PBS (1X) containing 4 mg/mL of dopamine hydrochloride (99%, Sigma), rinsed in PBS and used directly for cell probes preparation. For that, single-cells from 50 µL of diluted bacterial suspension (×100) were immobilized on the colloidal probes. Cell probes were then used to measure cells-biofilm interaction forces. All the force curves obtained were analyzed using Data Processing software from JPK Instruments (Bruker, USA). Adhesion force histograms were obtained by calculating the maximum adhesion force of the last peak for each curve. For each strain, experiments were repeated at least three times with cells coming from independent cultures.

### Statistical analysis

In this work, for each parameter (Biofilms thickness, average roughness, Young’s modulus and adhesion force), a statistical analysis was performed to compare the biofilms of the different strains. To do this, IBM Spss Statistic 25 and Microsoft Excel software were used to perform the ANOVA test. The test was done at the threshold of 5%, 1% and 0.1%.

## Results

### Pili influence the structural development of *L. lactis* biofilms

**Figure 1:**
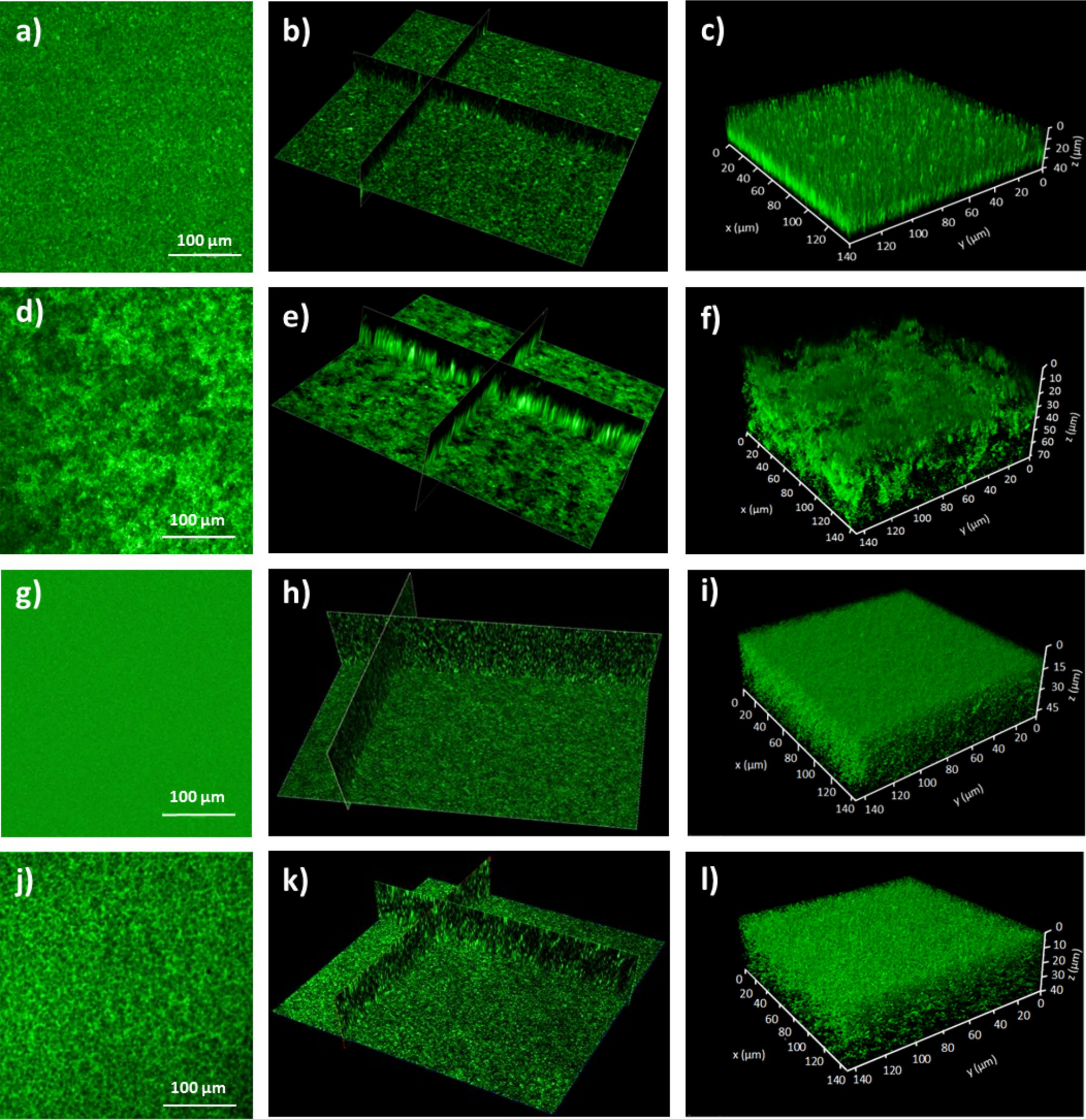
CLSM analyses of 24-h biofilms formed by *Lactococcus lactis* strains. (**a**–**c**) Biofilms of Pil^−^ VE17061, (**d**–**f**) Pil^++^ VE17176, (**g**–**i**) Pil^−^ Mub^−^ TIL1230 and (**j**–**l**) Pil^+^ Mub^+^ TIL448 were stained with Syto-9 dye from the *BacLight* and processed for CSLM. 2D views (**a**,**d**,**g**,**j**), cross-section of 3D volume image (**b**,**e**,**h**,**k**) and surface plot of 3D volume images (**c**,**f**,**i**,**l**) are presented for each strain.

To reproduce the result of Oxaran and *al.*^14^ demonstrating that *L. lactis* biofilms are, somehow, structured by the display of pili at the surface of lactococci cells, biofilms were obtained with IL1403 derivatives, Pil^−^ VE17061 and Pil^++^ VE17176 and they were analyzed by CSLM. In addition, to evaluate the role of pili in biofilm structuration, biofilms formed by the environmental strain TIL448 that displays both pili and Mub proteins (Pil^+^ Mub^+^ TIL448) and its control (Pil^−^ Mub^−^ TIL1230) were also analyzed. The results presented in Fig. 1 show that the biofilm of the Pil^−^ VE17061 control strain (Fig. 1a–c) exhibited a compact and uniform biofilm while the biofilm of Pil^++^ VE17176 was thicker and heterogeneous (Fig. 1d–f) thus confirming previous observations^14^. In addition, as expected, a compact structure was observed for the biofilm of the Pil^−^ Mub^−^ TIL1230 control strain (Fig. 1g–i) even more homogeneous than the biofilm of Pil^−^ VE17061 (Fig. 1g–i). Interestingly, despite the presence of pili, the biofilm of Pil^+^ Mub^+^ TIL448 strain (Fig. 1j–l) was more homogeneous than the biofilm of Pil^++^ VE17176 and less compact than the two Pil^−^ control strains.

The height of the biofilms was monitored by making cross sections on the different biofilms. Figure 1e showed that the structure of the Pil^++^ VE17176 biofilm was thicker than that of the control Pil^−^ VE 17061 strain (Fig. 1b). In contrast, the biofilms of the Pil^+^ Mub^+^ TIL448 (Fig. 1k) and Pil^−^ Mub^−^ TIL1230 vegetal strains (Fig. 1h) did not show such high structures. This could be due to the presence of additional surface proteins displayed in the Pil^+^ Mub^+^ TIL448 strain. The height of all biofilms was evaluated on the z-scale (Fig. 1c,f,i,l). The 3-D image of the biofilm of Pil^−^ VE17061 was obtained from 113 stacks corresponding to a thickness of 38.7 µm. The number of recorded stacks and the biofilm thicknesses were of 139 (47.2 µm), 116 (40.0 µm) and 206 (70.9 µm) for Pil^−^ Mub^−^ TIL1230, Pil^+^ Mub^+^ TIL448 and Pil^++^ VE17176 strains, respectively.

### AFM imaging reveals the topography of *L. lactis* biofilm surface

**Figure 2:**
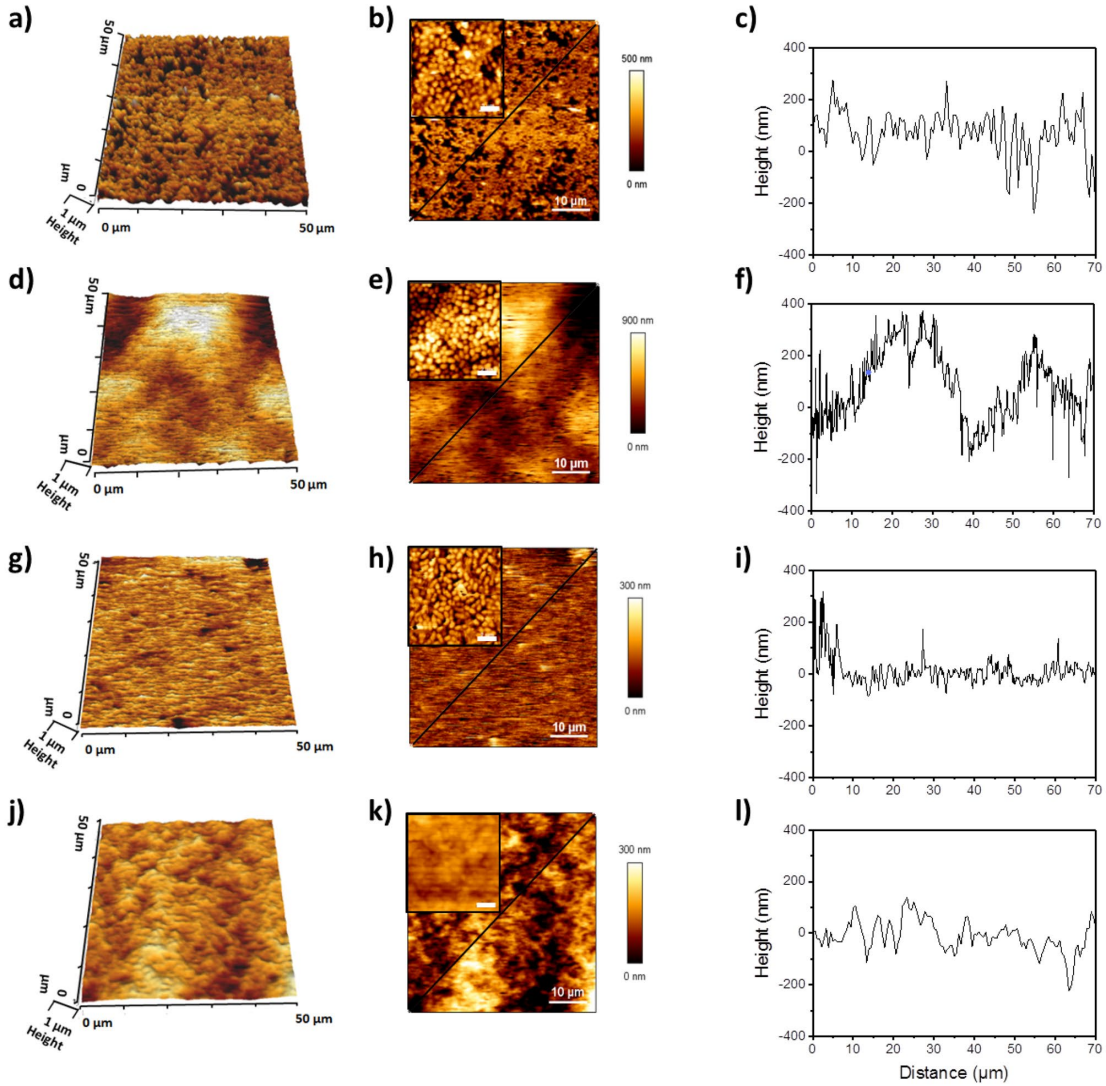
Topography analysis of 24-h *L*. *lactis* biofilms using AFM. High-resolution 3D images were recorded for an area of 50 µm × 50 µm with 16 × 16 pixels. Cross section profiles are indicated by the black lines. Studied strains: Pil^−^ VE17061 (**a**–**c**), Pil^++^ VE17176 (**d**–**f**), Pil^−^ Mub^−^ TIL1230 (**g**–**i**) and Pil^+^ Mub^+^ TIL448 (**j**–**l**). The scale bar in the insert b, e, h and k, corresponds to 2 µm.

Biofilm surface topography was obtained from AFM images recorded on areas of 50 × 50 µm. High-resolution images obtained from the scans as well as 2D and 3D micrographs are presented in Fig. 2. Cross sections made transversely were taken on these images according to the black line (Fig. 2c,f,i,l). The surface of the biofilms of Pil^−^ VE17061 (Fig. 2a–c) and Pil^−^ Mub^−^ TIL1230 (Fig. 2g–i) appeared to be flatter and more compact than that of Pil^++^ VE17176 and Pil^+^ Mub^+^ TIL448 biofilms (Fig. 2j–l). The biofilm of Pil^+^ Mub^+^ TIL448 showed dense micro-aggregate (Fig. 2j,l) compared to the biofilm of Pil^++^ VE17176 which exhibited large cell agglomerates (Fig. 2d,e), as could be seen also in the cross-section profiles showing large pics (Fig. 2f) corresponding to macro-aggregates. Cross section profiles of the two control strains (Fig. 2c,i) confirmed that the biofilm of Pil^−^ Mub^−^ TIL1230 was more compact and more homogeneous than that of Pil^−^ VE17061.

### Roughness analyses of *L. lactis* biofilms

**Figure 3:**
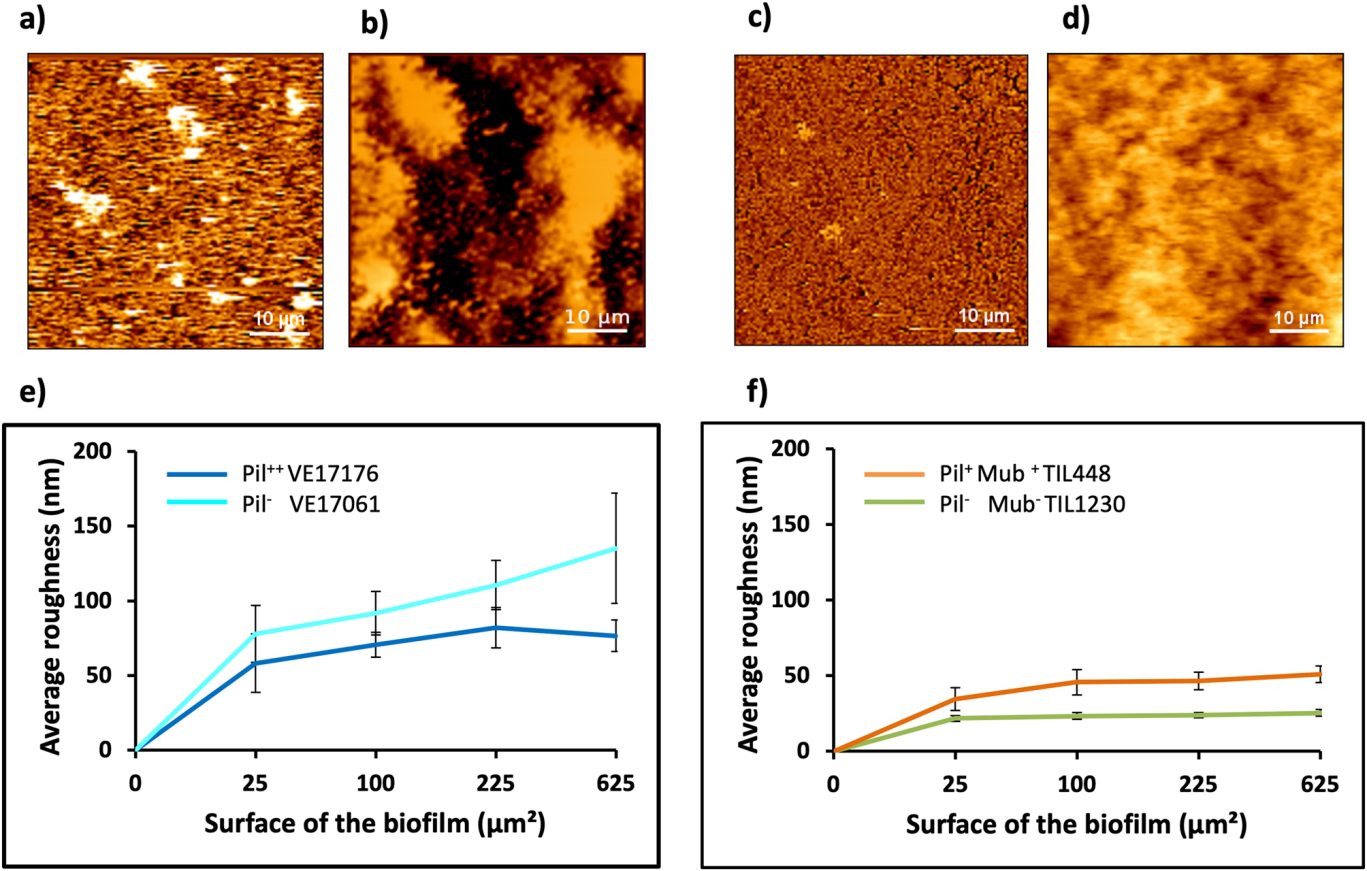
Roughness analyses of *L. lactis* biofilms using AFM technique. High resolution images of 24-h biofilms were recorded for Pil^−^ VE17061 (**a**), Pil^++^ VE17176 (**b**), Pil^−^ Mub^−^ TIL1230 (**c**) and Pil^+^ Mub^+^ TIL448 (**d**) strains. (**e**) and (**f**) showed the average roughness (Ra) measured on each biofilm for an area of 5 µm × 5 µm, 10 µm × 10 µm, 15 µm × 15 µm and 25 µm × 25 µm. The error bars in panel’s **e** and **f** denote three measurements from three independents biofilms.

The average roughness (Ra) at the biofilm surface was estimated from the topography AFM scans with high-resolution images (Fig. 3a–d) and analyzed for each strain. In each case, the roughness was measured on different areas of the surface on the images recorded as shown in Fig. 2. For the laboratory strains (Fig. 3e), the Ra varied from 58.2 ± 19.6 to 76.6 ± 10.4 nm for the Pil^−^ VE17061 biofilm and from 77.8 ± 19.1 to 135.1 ± 36.8 nm for the Pil^++^ VE17176 biofilm. For the vegetal strains, the control Pil^−^ Mub^−^ TIL1230 biofilms (Fig. 3f) displayed more homogeneous and smoother (21.6 ± 1.8 to 25.3 ± 2.2 nm) surface than all other biofilms thus confirming the previous results, and the Ra of the Pil^+^ Mub^+^ TIL448 biofilms was in the range of 34.5 ± 7.5 to 50.5 ± 5.5 nm. Clearly the biofilm surface obtained with the vegetal strains was smoother than those observed in laboratory strains. This could be due to the presence of other components of the extracellular matrix in addition to the pili, which makes the Pil^+^ Mub^+^ TIL448 biofilms more compact and smoother than the Pil^++^ VE17176 biofilms.

### Nanomechanical properties of the *L. lactis* biofilms surface

**Figure 4:**
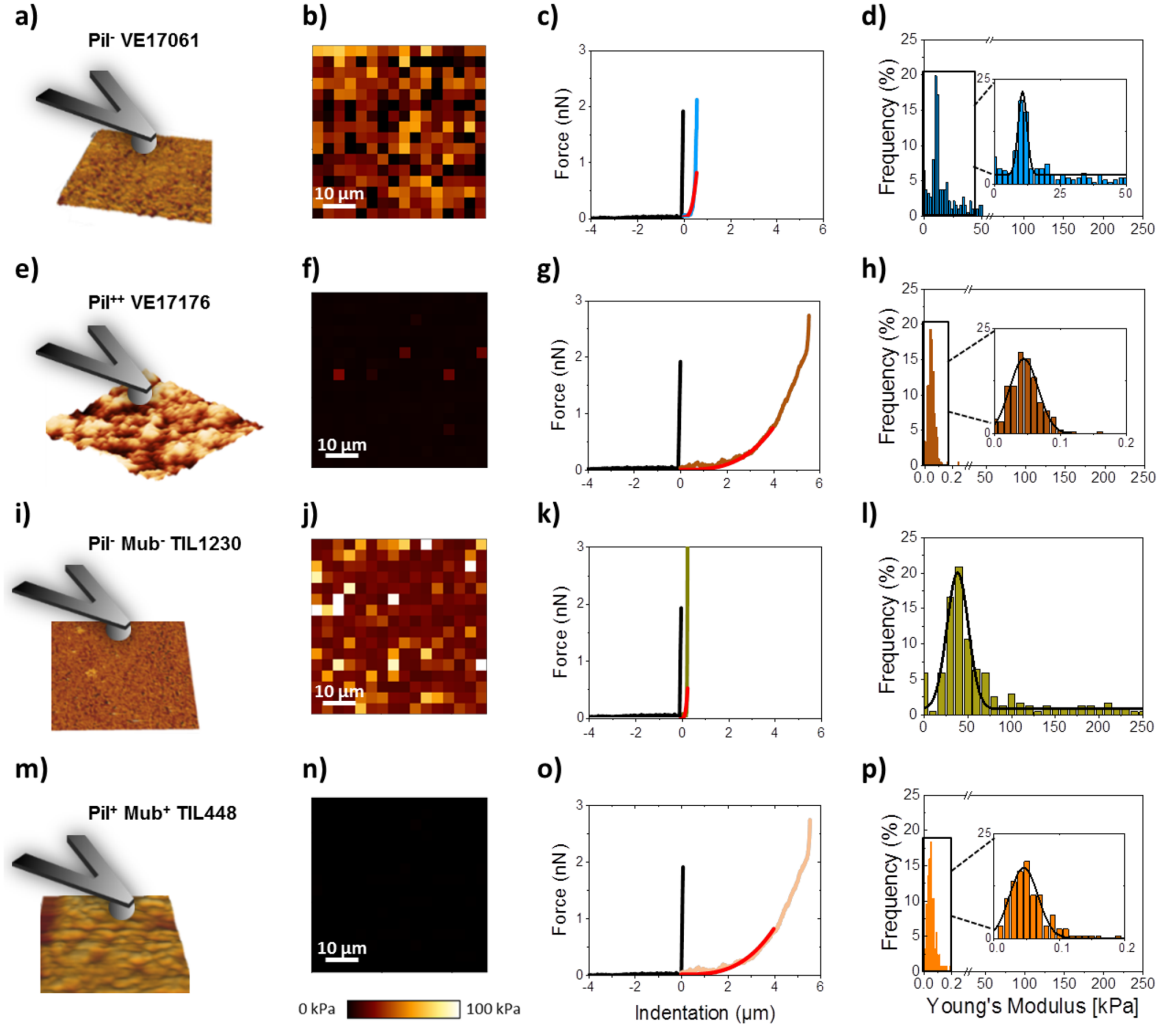
Nanomechanical properties of *L. lactis* biofilm surface. (**a**,**e**,**i**,**m**) Schematic representation of the measurement of the interactions between a colloidal probe (silica microsphere) and 24 h-biofilm. Statistical distribution of the Young’s modulus (**b**,**f**,**j**,**n**), elasticity maps (**c**,**g**,**k**,**o**) and force–indentation curves with theoretical model (red line) taken from the substrate (black line) and the biofilm of Pil^−^ VE17061 (blue line) (**d**), Pil^++^ VE17176 (brown line) (**h**), Pil^−^ Mub^−^ TIL1230 (green line) (**l**) and Pil^+^ Mub^+^ TIL448 (orange line) (**p**). All force-curves were recorded for an area of 50 µm × 50 µm corresponding to 16 × 16 pixels.

To investigate whether the display of pili at the cell surface affects the nanomechanical properties of the different *L. lactis* biofilms, force spectroscopy experiments^51^ were performed using spherical AFM probes (Fig. 4a,e,i,m). The elastic modulus of biofilms from at least three biofilms of piliated and control strains in each case were compared by recording 16 × 16 force curves for an area of 50 µm × 50 µm. The results obtained from one culture are represented in Fig. 4; the data corresponding to the other biofilms for the same strain respectively are presented in the supplementary material section (Figure S1). Figure 4b,f,j,n show the elastic maps for the biofilm of the four strains. On each map, each little square corresponds to one force curve. As expected, and shown in Fig. 4f,n, the maps obtained for piliated strains display dark contrast near to 0 kPa compared to the control strains maps showing heterogeneous contrast and high elastic modulus (Fig. 4b,j). This significant difference in elasticity between piliated and control strains (Table 3) was quantified by fitting the indentation curves with the Hertz model. The indentation curves were calculated by subtracting the cantilever deflection to the force–distance curves. The indentation is therefore the penetration depth of the probe into the biofilm. For the same applied force, the indentation in a soft material is higher than in a hard material. The comparison of the indentation curves recorded on the different biofilms, fitted to the Hertz model (plain red lines in Fig. 4c,g,k,o), clearly confirmed that the two piliated strains biofilms showed higher indentation, and therefore exhibited a much softer behavior than the control strains. These observations were confirmed by the quantitative analyses of the Young’s modulus distributions represented in the histograms: Fig. 4d,h,l,p. The Young’s modulus value of the Pil^−^ VE17061 biofilm varied from 4 to 50 kPa and was on the average of 11 ± 2 kPa (Fig. 4d). For the biofilm of Pil^−^ Mub^−^ TIL1230, the Young’s modulus ranged from 25 to 100 kPa with an average of 48 ± 12 kPa (Fig. 4l). In contrast, in piliated strains, the Young’s modulus values were lower: 0.05–0.20 kPa with an average of 0.04 ± 0.02 kPa for the Pil^++^ VE17176 (Fig. 4h) and 0.01–0.40 kPa with the average of 0.04 ± 0.02 kPa for the Pil^+^ Mub^+^ TIL448 (Fig. 4p) strains. These results indicated that the biofilms of piliated strains Pil^++^ VE17176 and Pil^+^ Mub^+^ TIL448 were softer than the biofilms of pili-devoid strains Pil^−^ VE17061 and Pil^−^ Mub^−^ TIL1230. The more compact biofilm of Pil^−^ Mub^−^ TIL1230 was stiffer than the biofilm of Pil^−^ VE17061 which may be due to the display of additional surface proteins in the Pil^−^ Mub^−^ TIL1230 strain^38^.

### Cell cohesiveness in *L. lactis* biofilms

**Figure 5:**
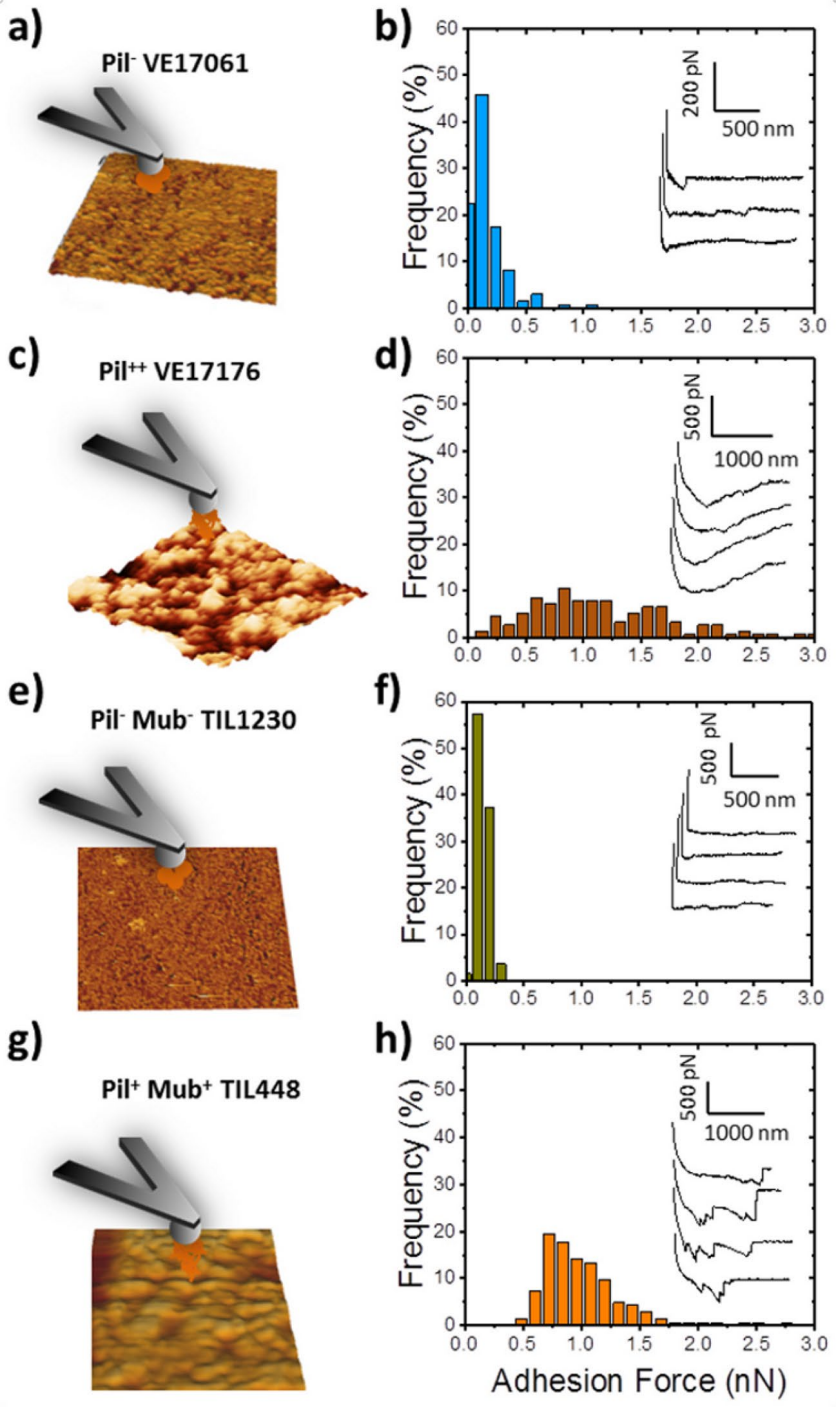
Quantification of the interactions between individual cells and 24 h-biofilms of *L. lactis*. (**a**,**c**,**e**,**g**) show measurement methods. (**b**,**d**,**f**,**h**) Adhesion force histograms and typical force curves obtained by recording force curves on 50 µm × 50 µm biofilm surface with 16 × 16 pixels in Pil^−^ VE17061 (**b**), Pil^++^ VE17176 (**d**), Pil^−^ Mub^−^ TIL1230 (**f**), and Pil^+^ Mub^+^ TIL448 (**h**) strains.

In order to demonstrate the influence of pili in the cohesive strength of the biofilms, we decided to quantify cell–biofilm interactions using colloidal probes functionalized with bacteria and brought in contact of the biofilm-interface (Fig. 5a,c,e,g). Force spectroscopy experiments were therefore conducted between cells on the probe and 24-h biofilms of *L. lactis*. The histograms showing the distribution of the adhesion forces recorded, as well as typical force–distance curves obtained in each case, are presented Fig. 5d,f,h. As it can be seen in Fig. 5b,f, the measurements between the two-control strains (Pil^−^ VE17061 and Pil^−^ Mub^−^ TIL1230) and their respective biofilms resulted in adhesion forces in the range of 0.12–0.25 nN. The adhesion forces for the two piliated strains, Pil^++^ VE17176 (Fig. 5d) and Mub^+^ TIL448 (Fig. 5h) were stronger, and ranged from 0.5 to 2 nN. The typical force curves observed for piliated strains showed multi-signatures that were more frequent for Pil^+^ Mub^+^ TIL448 because of the unfolding of several proteins (Mub among others) in addition to pili. These signatures were missing for pili-devoid strains. These results demonstrated that pili were clearly involved in the high adhesion of the cells in mature biofilm.

To facilitate the comparison between strains, the results concerning the thickness, average roughness, Young’s modulus and adhesion force values are gathered in a recapitulative table (Table 2). For the sake of transparency, we also gathered the results of the ANOVA test applied to the 4 different parameters for the 4 different strains (Table 3). Globally the strains devoid of pili displayed stiffer biofilms (4–100 kPa) and smoother surface than the piliated strains (Young Modulus around 0.04–0.1 kPa). The presence of others surface proteins does not seem to affect the stiffness but make the biofilm smoother and more compact, as observed in the environmental strain. Finally, we demonstrated the role of pili and other surface proteins in the biofilms cohesiveness by testing the homotypic adhesion of bacteria to the biofilm surface.

**Table 2:**
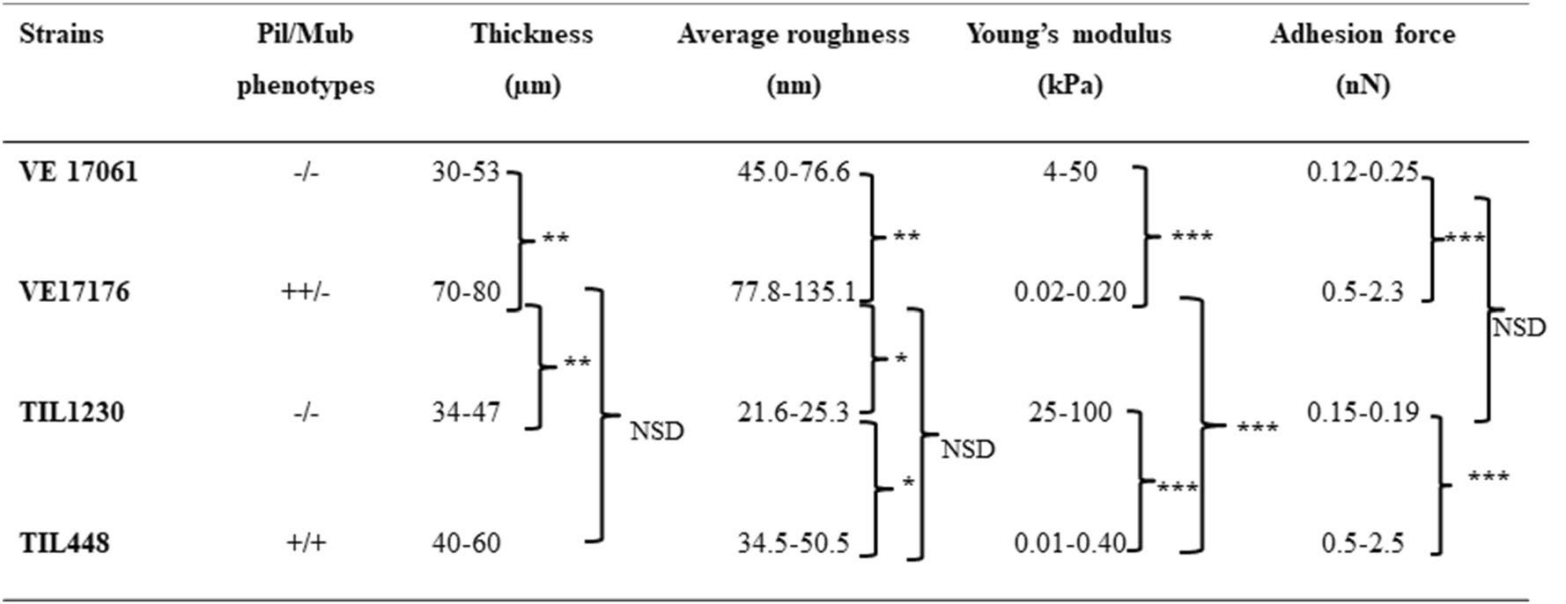
Summary table of results: biofilm thickness, average roughness, Young’s modulus and adhesion force are reported for the four studied strains.

For each parameter, a statistical analysis was performed by the ANOVA test at the threshold of 5% (*), 1% (**) and 0.1% (***). NSD: no significant difference.

**Table 3:** Comparaison of the average values obtained for one strain against those obtained for another one.

Comparaison of strains	Thickness	Average roughness	Young modulus	Adhesion force
Pil^−^ VE 17061/Pil^++^ VE17176	**	**	***	***
Pil^−^ VE 17061/Pil^−^ Mub^−^ TIL1230	NSD	*	NSD	NSD
Pil^−^ VE 17061/Pil^+^ Mub^+^ TIL448	NSD	NSD	***	***
Pil^++^ VE17176/Pil^−^ Mub^−^ TIL1230	**	***	***	***
Pil^++^ VE17176/Pil^+^ Mub^+^ TIL448	**	**	NSD	NSD
Pil^+^ Mub^+^ TIL448/Pil^−^ Mub^−^ TIL1230	NSD	*	***	***

For this, the ANOVA test for each parameter (thickness, average roughness, Young modulus and Adhesion force) was used with threshold of 5% (*), 1% (**) and 0.1% (***). NSD: no significant difference.

## Discussion

In the present work, we demonstrate the role of *L. lactis* pili in biofilm architecture and nanomechanical properties. Confocal scanning laser microscopy (CSLM) and AFM imaging were used to examine the structure and the topography of biofilms of different *L. lactis* strains. CSLM image of 24-h biofilms revealed that the biofilm obtained with the Pil^−^ VE17061 strain was compact and uniform whereas the Pil^++^ VE17176 strain formed a heterogeneous biofilm with dense aggregates and aerial structure. These results confirmed the previous works carried out on the same strains^14^. Similar observations on the role of pili in biofilm architecture are in line with the results obtained on the hyperpiliated mutant’s of *Pseudomonas aeroginosa*^52^. Indeed, using CLSM it was shown that the mutants devoid of type IV pili did not form microcolonies during the biofilm formation and leads to the formation of a homogeneous biofilm structure.

To go one step further in the complexity of the system, we shifted to a couple of natural vegetal isolates, the Pil^+^ Mub^+^ TIL448 strain harboring both pili and Mub-domain proteins at its surface and the Pil^−^ Mub^−^ TIL1230 derivative devoid of those two surface determinants^25,38^. As illustrated in Fig. 4, the biofilm obtained for the Pil^+^ Mub^+^ TIL448 strain was surprisingly homogeneous despite the presence of pili. However, it appeared to be less compact than the biofilm of the control strain (Pil^−^ Mub^−^ TIL1230) pointing out the influence of pili. These results were unexpected for the strain Pil^+^ Mub^+^ TIL448 as we rather expected a biofilm with a structure close to that of the laboratory piliated strain Pil^++^ VE17176. This discrepancy could be attributed to several hypotheses (i) the Mub-proteins may play a role in the more compact structure of Pil^+^ Mub^+^ TIL 448 biofilms, (ii) Pil^+^ Mub^+^ TIL 448 might produce other surface proteins^38^ that could increase the adhesion of the bacteria within the biofilm, (iii) the Pil^+^ Mub^+^ TIL 448 strain produced high number of exopolysaccharides, compared to the laboratory strains, that could be involved in the building of a homogenous and compact biofilm matrix. In addition, it is highly probable that the expression level of the pilus biosynthesis genes in the natural Pil^+^ Mub^+^ TIL448 strain is lower than that of the laboratory Pil^++^ VE17176 strain in which the whole *pil* operon and the *srtA* gene involved in cell–wall attachment of the pilus, are both overexpressed at high level^14^. This could also explain the difference of the thickness of the two biofilms. As a result, the Pil^++^ VE17176 strain exhibits an aerial biofilm with macro-aggregates as observed by AFM high-resolution images and cross-section profiles (Fig. 2d–f). These characteristics of the Pil^++^ VE17176 biofilm have also been correlated with a high surface roughness compared to that of the Pil^−^ VE17061 biofilm and to those obtained with the environmental strains that were flat and smooth (Fig. 3).

Interestingly, the experiments performed in the environmental strain TIL448 shed light on the relationship between pili, Mub-proteins and extracellular polymeric substances at the biofilm level. The interactions discovered between pili and Mub-proteins^25^, at the molecular scale and the significant presence of a diversity of proteins on the surface of strain TIL448^38^ seems to have an impact at the biofilm scale and lead to the production of a denser and more compact biofilm than in the absence of Mub-protein and pili (Pil^−^ Mub^−^ TIL1230).

The relationship between pili and other surface proteins at the biofilm scale is increasingly being studied. Recently, Wang and coworkers^53^ investigated, using AFM, the role of the interactions between type 3 fimbriae and the polysaccharidic capsule of *Klebsiella pneumoniae* in biofilm formation. They concluded that the type 3 fimbriae help maintaining the fluidity of the polysaccharidic capsule that is involved in the biofilm organization. In another study^54^, it has been shown that the curli-like pili produced by Salmonella, and the other components of the extracellular matrix played an important role in biofilm morphology and curli seem to be indispensable for the formation of cell aggregates rather than the other components of the matrix^51^.

AFM has been extensively used during the last decades to study single bacteria or even single molecules at the bacterial surface. However, studies at the scale of the biofilm are uncommon and very few have been developed on hydrated biofilms. In this work, it should be noted that AFM imaging and force spectroscopy were carried out in an aqueous medium because it has been shown that dried biofilms change in morphology, roughness or adhesion forces when compared to moist biofilms^55^. Drying biofilms before imaging permits to obtain high-resolution images of the microbial interfaces^56,57^, but the experimental conditions are quite different from the biological reality of a biofilm that is only formed in aqueous solution.

Next to the fact that AFM allows to obtain high-resolution images and to compare the topology and roughness of biofilm surfaces in aqueous environment, it can also be used to evaluate biofilms elastic behavior. In the present work, we have also measured the mechanical properties of hydrated *L. lactis* biofilms and their relationship with biofilm architecture. We clearly demonstrate the influence of pili in the mechanical properties of biofilms. As shown in Fig. 4 and in supplementary material (Figure S1), the biofilms of piliated strains exhibited a dramatic reduction of their Young’s modulus (up to a thousand times) compared to control strains devoid of pili (in average 0.04 ± 0.02 kPa for piliated strains versus 11 ± 2 kPa and 48 ± 12 kPa for Pil^−^ VE17061 and Pil^−^ Mub^−^ TIL1230 control stains, respectively). This demonstrated a dramatic softening of the biofilms related to the display of pili and to the aerial structuration that they cause. To formulate the Young's modulus values differently, it can be said that, for the same applied force of 0.5 nN, the probe penetrates 20 nm into the biofilms of non-pilled strains and about 4000 nm into the biofilms of piliated strains. To further confirm this substantial significant difference (Table 3), we compared the spring constant of each biofilm by measuring the slope of the indentation curves. The results confirmed that the biofilms of piliated strains were much softer (0.23 ± 0.13 to 0.42 ± 0.21 nN/µm) than the biofilm of control strains that exhibited spring constant ranging from 7.25 ± 3.5 to 38.01 ± 13.01 nN/µm (supplementary material, Figure S2). These results demonstrated the key role of pili in the elasticity of biofilms whatever the organization of the cells.

The potential role of *L. lactis* pili in cell adhesion within biofilms was investigated by measuring the interaction between bacterial cells attached to the colloidal probe and the biofilm-interface. The measured adhesion forces were higher for the piliated strains, in the range of 0.5 to 2 nN, than for the pili-devoid cells with values of 0.12 to 0.25 nN. The high adhesion level obtained for piliated cells is most likely due to the elasticity of biofilm surfaces, suggesting that the pili played key role in cell–cell interactions in biofilm formation. Other studies have shown that the production of extracellular polymeric substances could also be involved in cell cohesion^40,58^. This measure of interaction between cells and a biofilm surface are scarce in the literature. It is due to the difficulty to perform experiments on a hydrated biofilm. However, the biofilm ability to interact with other cells is the key information to evaluate for example, the capacity of a beneficial biofilm to trap pathogenic bacteria^58^.

## Conclusion

This work reports on the analysis of the architecture, the structuration, the biomechanics and the cohesiveness of hydrated biofilms of *L. lactis* strains expressing or not, pili, Mub proteins and other surface proteins. We demonstrate that probing a hydrated biofilm by AFM is possible and provide valuable data on its structure.

In summary, the use of CSLM and AFM made it possible to analyze and study the role of pili in the architecture and surface morphology of *L. lactis* hydrated biofilms. We were able to demonstrate on one hand that pili were involved in the structuration, the biomechanics and the cohesiveness of *L. lactis* biofilms, and on the other hand, that other surface proteins were involved in the structuration but have no role nor in the biomechanics, nor on the biofilm cohesiveness. The piliated biofilms were a thousand times softer than biofilms of the control strains proving the crucial role of pili in biofilm biomechanics. The higher adhesion forces between the cells attached on colloidal probe and the biofilm of piliated strains proved that pili were responsible for a global increase of the biofilm cohesiveness. Their interactions with other surface proteins result in a modification of the structure, architecture and surface roughness but do not modify the biofilm biomechanical properties and cohesiveness. This analysis of biofilm biomechanical properties and cohesiveness would contribute, in the future, to elucidate the influence of pili in the resistance of *L. lactis* biofilms to hydrodynamic flow.

Altogether, this work showing that pili and surface proteins can shape the biofilms of *L. lactis* may open new doors in probiotic and biotechnological applications in which this bacteria is used. In probiotic issues, for example, it has been shown in lactobacilli that the biofilm mode of life boosts the biological activity mediated by lactobacilli^59,60^. Also, it can be hypothesized that more stable biofilms of *L. lactis* might increase the persistence and retention time of the bacteria in the gastrointestinal tract and thus increase the beneficial effects mediated by this bacteria. In biotechnological issues including food processing, *L. lactis* biofilms are involved in a number of applications^15^. Therefore, they are proposed as candidates in the growing-interest field of bioprotective biofilms able to prevent or hamper the development of pathogenic and spoilage bacteria on (a) biotic surfaces present in the food chain. In this field, the possibility to modulate the spatial structure of biofilms is of great interest to optimize their ability to trap unwanted bacteria In addition, the ability to modulate biofilm robustness will be important to allow their resistance to shear flow likely encountered in food industry environments.

## Supplementary Information

Supplementary Figures
