# Supplementary material for: Pili and other surface proteins influence the structure and the nanomechanical properties of Lactococcus lactis biofilms
Source: Sci Rep. 2021 Mar 1;11:4846. doi: 10.1038/s41598-021-84030-1 (PMC7921122; doi:10.1038/s41598-021-84030-1)
Supplement: Supplementary file 1 — Supplementary Figures [file 41598_2021_84030_MOESM1_ESM.docx]

**Supplementary material:**

**Pili and other surface proteins influence the structure and the nanomechanical properties of *Lactococcus lactis* biofilms**

Ibrahima DRAMÉ^1,2^, Christine LAFFORGUE^1^, Cécile FORMOSA-DAGUE^1^, Marie-Pierre CHAPOT-CHARTIER^3^, Jean-Christophe PIARD^3^, Mickaël CASTELAIN^1^, Etienne DAGUE*^2^

1. TBI, Université de Toulouse, INSA, INRAE, CNRS, Toulouse, France
2. LAAS- CNRS, Université de Toulouse CNRS, Toulouse, France
3. Université Paris-Saclay, INRAE, AgroParisTech, Micalis Institute, 78350, Jouy-en-Josas, France.

* Corresponding author: Etienne Dague: edague@laas.fr

**Supplementary material 1:** **Nanomechanical properties of *L. lactis* biofilm surface.
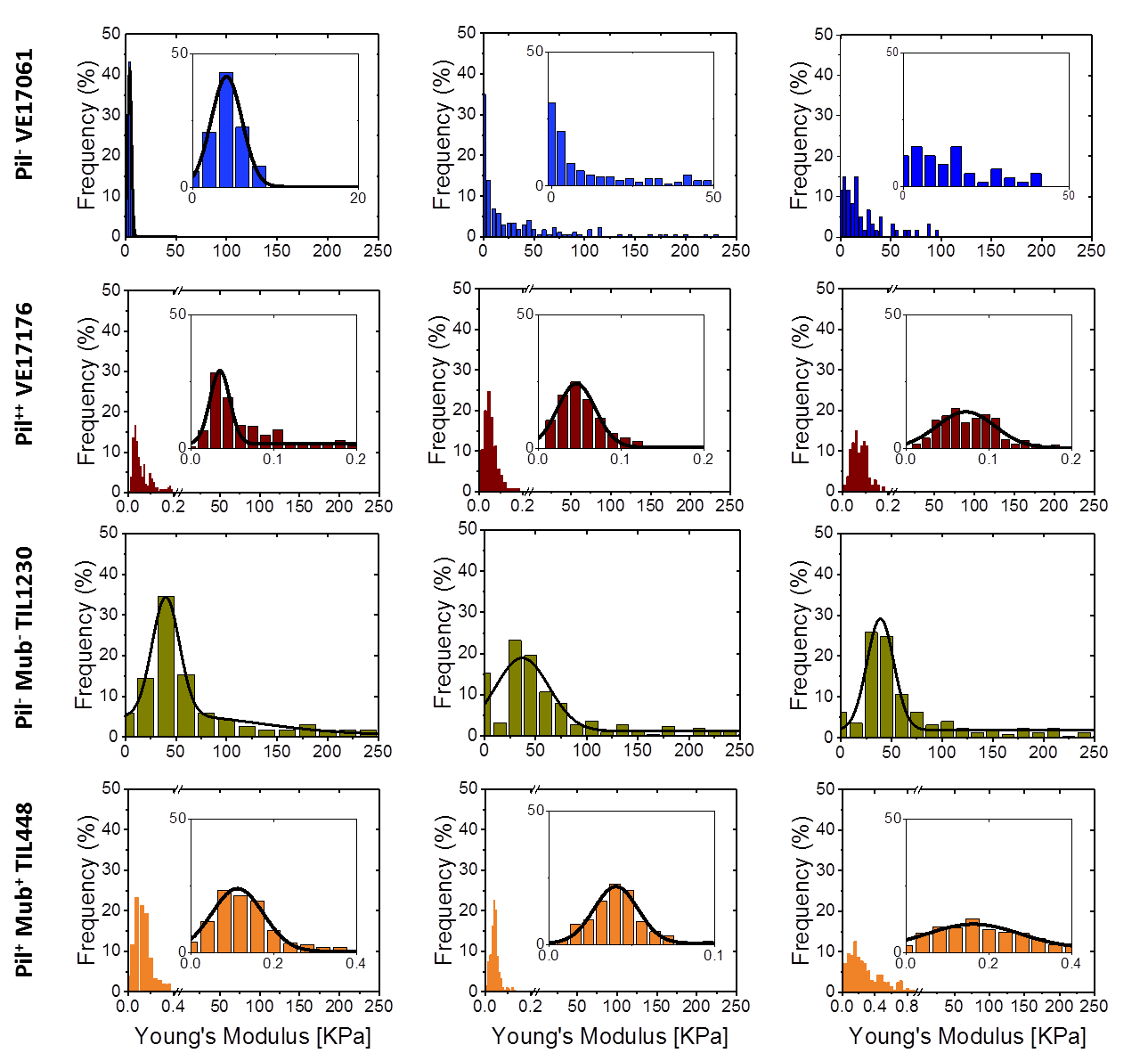
**

**Figure S1:** Statistical distributions of Young’s modulus values of the different strains of *L. lactis* are presented. For each strain, four independent experiments were recorded. All force-curves were recorded for an area of 50 µm × 50 µm with 16 × 16 pixels.

**Supplementary material 2:** **The stiffness constants of the biofilms surface of different strains of *L. Lactis*.**


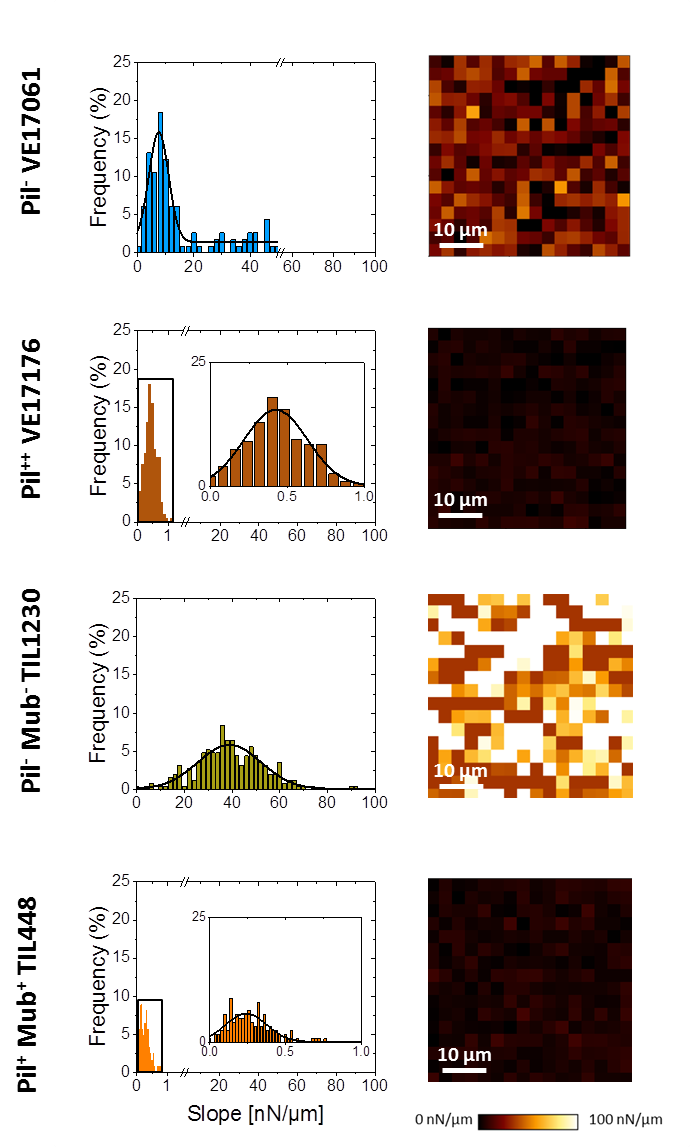


**Figure S2:** The slope of the force-curves and the elastic maps recorded from an area of 50 µm × 50 µm on the Pil^-^ VE17061, the Pil^++^ VE17176, the Pil^-^ Mub^-^ TIL1230 and the Pil^+^ Mub^+^ TIL448**.**

**Supplementary material 3: Interaction between individual cells on coated colloidal probe and biofilm of *L. lactis*.**

**
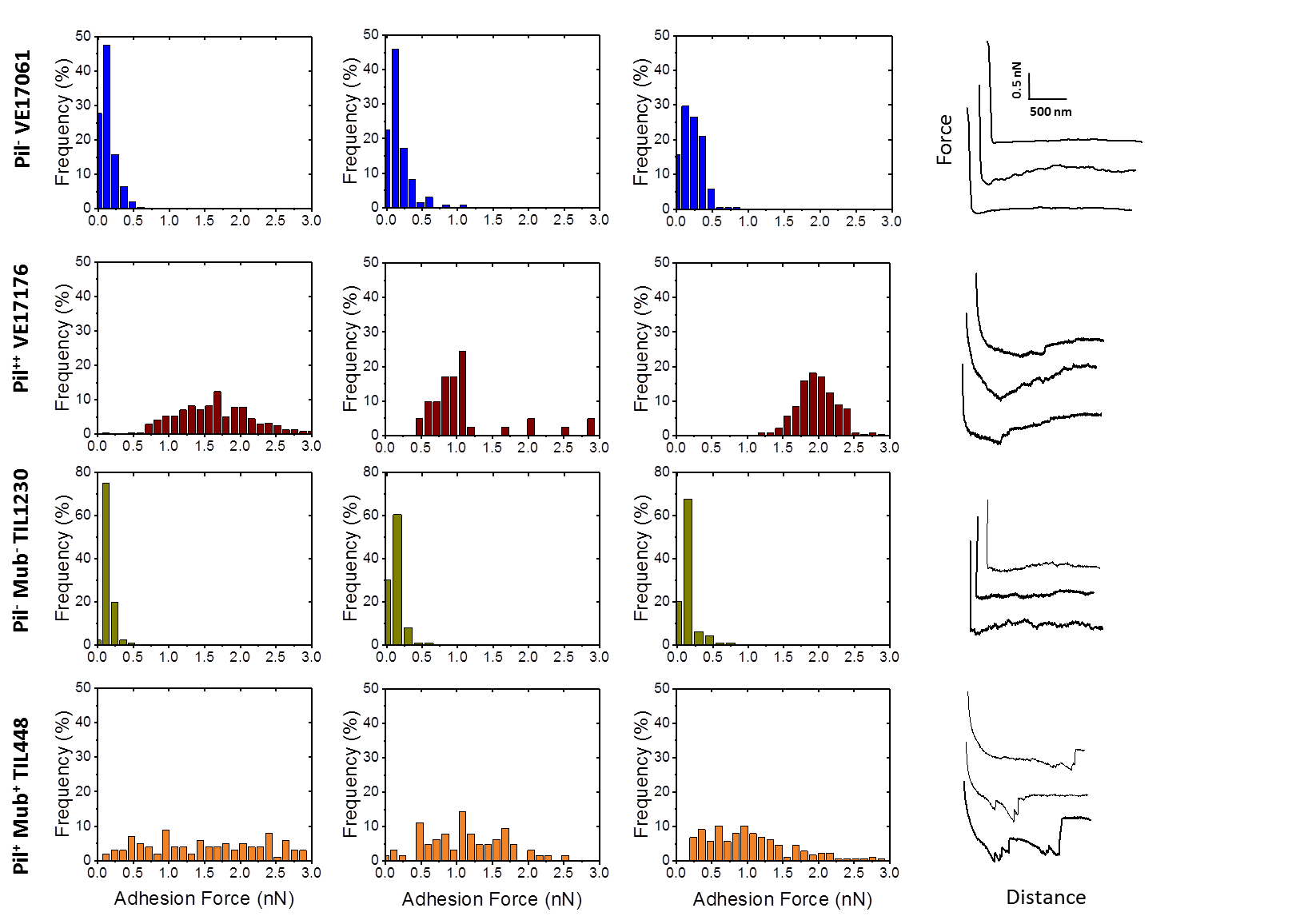
**

**Figure S3:** Adhesion force histograms and typical force curves obtained by recording force curves on 50 µm × 50 µm biofilm surfaces with 16 x 16 pixels in Pil- VE17061 (b), Pil++ VE17176, Pil- Mub- TIL1230, and Pil+ Mub+ TIL448 strains. For each strain, four independent cultures were represented.
